# Precise Point Positioning Using Triple GNSS Constellations in Various Modes

**DOI:** 10.3390/s16060779

**Published:** 2016-05-28

**Authors:** Akram Afifi, Ahmed El-Rabbany

**Affiliations:** Department of Civil Engineering, Ryerson University, Toronto, ON M5B 2K3, Canada; rabbany@ryerson.ca

**Keywords:** precise point positioning, GPS, Galileo, BeiDou

## Abstract

This paper introduces a new dual-frequency precise point positioning (PPP) model, which combines the observations from three different global navigation satellite system (GNSS) constellations, namely GPS, Galileo, and BeiDou. Combining measurements from different GNSS systems introduces additional biases, including inter-system bias and hardware delays, which require rigorous modelling. Our model is based on the un-differenced and between-satellite single-difference (BSSD) linear combinations. BSSD linear combination cancels out some receiver-related biases, including receiver clock error and non-zero initial phase bias of the receiver oscillator. Forming the BSSD linear combination requires a reference satellite, which can be selected from any of the GPS, Galileo, and BeiDou systems. In this paper three BSSD scenarios are tested; each considers a reference satellite from a different GNSS constellation. Natural Resources Canada’s GPSPace PPP software is modified to enable a combined GPS, Galileo, and BeiDou PPP solution and to handle the newly introduced biases. A total of four data sets collected at four different IGS stations are processed to verify the developed PPP model. Precise satellite orbit and clock products from the International GNSS Service Multi-GNSS Experiment (IGS-MGEX) network are used to correct the GPS, Galileo, and BeiDou measurements in the post-processing PPP mode. A real-time PPP solution is also obtained, which is referred to as RT-PPP in the sequel, through the use of the IGS real-time service (RTS) for satellite orbit and clock corrections. However, only GPS and Galileo observations are used for the RT-PPP solution, as the RTS-IGS satellite products are not presently available for BeiDou system. All post-processed and real-time PPP solutions are compared with the traditional un-differenced GPS-only counterparts. It is shown that combining the GPS, Galileo, and BeiDou observations in the post-processing mode improves the PPP convergence time by 25% compared with the GPS-only counterpart, regardless of the linear combination used. The use of BSSD linear combination improves the precision of the estimated positioning parameters by about 25% in comparison with the GPS-only PPP solution. Additionally, the solution convergence time is reduced to 10 minutes for the BSSD model, which represents about 50% reduction, in comparison with the GPS-only PPP solution. The GNSS RT-PPP solution, on the other hand, shows a similar convergence time and precision to the GPS-only counterpart.

## 1. Introduction

Global navigation satellite systems (GNSS) precise point positioning (PPP) has proven to be capable of providing positioning accuracy at the sub-decimeter and decimeter levels in static and kinematic modes, respectively. PPP differs from differential positioning methods in that differential techniques require access to GNSS observations from one or more reference stations with precisely known coordinates. This provides an advantage for PPP over differential methods, as only a single receiver is required at the user’s end. Consequently, the spatial operating range limit of differential techniques is overcome through PPP. However, a major disadvantage of PPP in comparison with differential GNSS techniques is that a relatively long time is required for the solution to converge to sub-decimeter level. This is mainly due to the existence of unmodelled residual errors Generally, PPP solution accuracy and convergence time are influenced by the ability to mitigate all potential error sources in the system. PPP essentially relies on the availability and use of precise satellite products, namely orbital and clock corrections. At present, the IGS-MGEX provides precise satellite orbital and clock corrections and the satellite hardware delays for all GNSS constellations [1].

Several comprehensive studies have been published on the accuracy and convergence time of un-differenced GPS PPP solution [2,3,4,5,6,7,8,9,10,11]. However, a drawback of a single GNSS constellation such as GPS is the limited number of visible satellites in urban areas. The addition of other GNSS systems, such as Galileo and BeiDou, offers more visible satellites to users, which in turn enhances the satellite geometry and is expected to improve the overall positioning solution [8]. This in turn makes the PPP solution more feasible, especially in urban areas. However, combining the observations of multi-GNSS constellations comes at the expense of introducing additional biases to the observation mathematical models. These include the GPS to Galileo time offset, GPS to BeiDou time offset and the hardware delays of both Galileo and BeiDou. Recently, [7] showed that combining the un-differenced GPS and Galileo observations in a PPP model improves the solution convergence time by about 25%, in comparison with the GPS-only counterpart. [12,13,14,15,16] combined the GPS/BeiDou observations in a PPP model, which reduced the convergence time by 20% in comparison with the GPS-only PPP solution. A quad-constellation (GPS, Galileo, BeiDou, and Glonass) PPP model was developed in [17,18,19,20] and its performance was assessed in static and kinematic modes. Their test results showed improvement in both of the PPP solution precision and convergence time. However, those studies were limited to the post-processing mode.

Recently, the focus of PPP has shifted to the determination of an accurate solution in real time. The advent of the IGS real-time Pilot Project in 2007 led to the development of real-time clock and orbit streams [4,21]. At present, the IGS real-time service provides different types of clock corrections, namely IGS01/IGC01, IGS02, and IGS03 [22,23]. The development of these correction streams allows the user to determine an accurate real-time PPP solution anywhere in the world. The availability of RT-PPP solution elevates PPP as a potentially viable alternative to differential GNSS techniques, in particular the highly productive and widely used real-time kinematic (RTK) solution. However, the long convergence time remains the main disadvantage of PPP [24,25,26]. 

This paper develops a triple-constellation GNSS (GPS, Galileo, and BeiDou) PPP model, which rigorously accounts for all errors and biases, including the additional biases introduced as a result of combining the observations of different GNSS constellations. These additional biases are lumped together into a new unknown parameter, which is referred to as the inter-system bias in our PPP model. In addition, the receiver differential hardware delays are lumped to the receiver clock error. The hydrostatic component of the tropospheric zenith path delay is modelled through the Hopfield model, while the wet component is considered as an additional unknown parameter [7,27]. All remaining errors and biases are accounted for using existing models as shown in [28]. The developed PPP model employs GPS L1/L2, Galileo E1/E5a, and BeiDou B1/B2 signals in dual-frequency ionosphere-free linear combinations. Sequential least-squares estimation technique is used to obtain the best estimates, in the least-squares sense, for the unknown parameters. It is shown that the convergence time of the un-differenced post-processed GNSS PPP solution is improved by about 25%, in comparison with the GPS-only counterpart. On the other hand, the use of BSSD linear combinations in post-processing mode improves the precision of the estimated positioning parameters by about 25% in comparison with the GPS-only counterpart. Additionally, the solution convergence time is reduced to 10 min BSSD model is used, which represents about 50% improvement in comparison with the GPS-only PPP solution. Moreover, the GNSS RT-PPP solution shows a similar convergence time and positioning precision to the GPS-only counterpart. In all cases, the inter-system bias parameter is found to be essentially constant over the observation time span (one hour) and is receiver-dependent.

## 2. Un-Differenced Post-Processing PPP Models

Traditionally, PPP has been carried out using dual-frequency ionosphere-free linear combinations of carrier-phase and pseudorange GPS measurements. Equations (1) to (6) show the ionosphere-free linear combinations of GPS, Galileo, and BeiDou observations [29,30].

(1) $$P_{G_{IF}}=\rho_{G}+c[dt_{rG}-dt^{s}]+c{\left[\alpha d_{P1}-\beta d_{P2}\right]}_{r}+c{\left[\alpha d_{P1}-\beta d_{P2}\right]}^{s}+T_{G}+\varepsilon_{PG_{IF}}$$

(2) $$P_{E_{IF}}=\rho_{E}+c[dt_{rG}-GGTO-dt^{s}]+c{\left[\alpha d_{E1}-\beta d_{E5a}\right]}_{r}+c{\left[\alpha d_{E1}-\beta d_{E5a2}\right]}^{s}+T_{E}+\varepsilon_{E_{IF}}$$

(3) $$P_{B_{IF}}=\rho_{B}+c[dt_{rG}-GB-dt^{s}]+c{\left[\alpha d_{B1}-\beta d_{B2}\right]}_{r}+c{\left[\alpha d_{B1}-\beta d_{B2}\right]}^{s}+T_{B}+\varepsilon_{B_{IF}}$$

(4) $$\Phi_{G_{IF}}=\rho_{G}+c[dt_{rG}-dt^{s}]+c{\left[\alpha \delta_{L1}-\beta \delta_{L2}\right]}_{r}+c{\left[\alpha \delta_{L1}-\beta \delta_{L2}\right]}^{s}+T_{G}+N_{G_{IF}}+\phi_{r0_{G_{IF}}}+\phi_{0_{G_{IF}}}^{s}+\varepsilon_{\Phi G_{IF}}$$

(5) $$\Phi_{E_{IF}}=\rho_{E}+c[dt_{rG}-GGTO-dt^{s}]+c{\left[\alpha \delta_{E1}-\beta \delta_{E5a}\right]}_{r}+c{\left[\alpha \delta_{E1}-\beta \delta_{E5a}\right]}^{s}+T_{E}+N_{E_{IF}}+\phi_{r0_{E_{IF}}}+\phi_{0_{E_{IF}}}^{s}+\varepsilon_{\Phi E_{IF}}$$

(6) $$\Phi_{B_{IF}}=\rho_{B}+c[dt_{rG}-GB-dt^{s}]+c{\left[\alpha \delta_{B1}-\beta \delta_{B2}\right]}_{r}+c{\left[\alpha \delta_{B1}-\beta \delta_{B2}\right]}^{s}+T_{B}+N_{B_{IF}}+\phi_{r0_{B_{IF}}}+\phi_{0_{B_{IF}}}^{s}+\varepsilon_{\Phi B_{IF}}$$

where the subscripts *G*, *E*, and *B* refer to the GPS, Galileo, and BeiDou satellite systems, respectively; $P_{G_{IF}}$, $P_{E_{IF}}$, and $P_{B_{IF}}$ are the ionosphere-free pseudoranges in meters for GPS, Galileo, and BeiDou systems, respectively; $\Phi_{G_{IF}}$, $\Phi_{E_{IF}}$, and $\Phi_{B_{IF}}$ are the ionosphere-free carrier phase measurements in meters for GPS, Galileo, and BeiDou systems, respectively; *GGTO* is the GPS to Galileo time offset; *GB* is the GPS to BeiDou time offset; *ρ* is the true geometric range from receiver at reception time to satellite at transmission time in meter; *dt_r_*, *dt^s^* are the clock errors in seconds for the receiver at signal reception time and the satellite at signal transmission time, respectively; $d_{P1}{}_{r}$, $d_{P2}{}_{r}$, $d_{E1}{}_{r}$, $d_{E5a}{}_{r}$, $d_{B1}{}_{r}$, $d_{B2}{}_{r}$ are frequency-dependent code hardware delays for the receiver at reception time in seconds; $d_{P1}{}^{S}$, $d_{P2}{}^{S}$, $d_{E1}{}^{S}$, $d_{E5a}{}^{S}$, $d_{B1}{}^{S}$, $d_{B2a}{}^{S}$ are frequency-dependent code hardware delays for the satellite at transmission time in seconds; $\delta_{L1r}$, $\delta_{L2r}$, $\delta_{E1r}$, $\delta_{E5ar}$, $\delta_{B1r}$, $\delta_{B2r}$ are frequency-dependent carrier-phase hardware delays for the receiver at reception time in seconds; $\delta_{L1}{}^{S}$, $\delta_{L2}{}^{S}$, $\delta_{E1}{}^{S}$, $\delta_{E5a}{}^{S}$, $\delta_{B1}{}^{S}$, $\delta_{B2}{}^{S}$ are frequency-dependent carrier-phase hardware delays for the satellite at transmission time in seconds; *T* is the tropospheric delay in meter; $N_{G_{IF}}$, $N_{E_{IF}}$, $N_{B_{IF}}$ are the ionosphere-free linear combinations of the ambiguity parameters for both GPS, Galileo, and BeiDou carrier-phase measurements in meters, respectively; $\phi_{r0_{G_{IF}}}$, $\phi_{0_{G_{IF}}}^{S}$, $\phi_{r0_{E_{IF}}}$, $\phi_{0_{E_{IF}}}^{S}$, $\phi_{r0_{B_{IF}}}$, $\phi_{0_{B_{IF}}}^{S}$ are ionosphere-free linear combinations of frequency-dependent initial fractional phase biases in the receiver and satellite channels for both GPS, Galileo, and BeiDou in meters, respectively; *c* is the speed of light in vacuum in meter per second; $\varepsilon_{P_{IF}}$, $\varepsilon_{E_{IF}}$, $\varepsilon_{\Phi G_{IF}}$, $\varepsilon_{\Phi E_{IF}}$, $\varepsilon_{B_{IF}}$, $\varepsilon_{\Phi B_{IF}}$ are the ionosphere-free linear combinations of the relevant noise and un-modeled errors in meter; $\alpha_{G}$, $\beta_{G}$, $\alpha_{E}$, $\beta_{E}$, $\alpha_{B}$, $\beta_{B}$ are the ionosphere-free linear combination coefficients for GPS, Galileo, and BeiDou which are given, respectively, by: $\alpha_{G}=\frac{f_{1}^{2}}{f_{1}^{2}-f_{2}^{2}}$, $\beta_{G}=\frac{f_{2}^{2}}{f_{1}^{2}-f_{2}^{2}}$, $\alpha_{E}=\frac{f_{E1}^{2}}{f_{E1}^{2}-f_{E5a}^{2}}$, $\beta_{E}=\frac{f_{E5a}^{2}}{f_{E1}^{2}-f_{E5a}^{2}}$, $\alpha_{B}=\frac{f_{B1}^{2}}{f_{B1}^{2}-f_{B2}^{2}}$, $\beta_{B}=\frac{f_{B2}^{2}}{f_{B1}^{2}-f_{B2}^{2}}$. where *f_1_* and *f_2_* are GPS L_1_ and L_2_ signals frequencies; *f_E1_* and *f_E5a_* are Galileo E_1_ and E_5a_ signals frequencies; *f_B1_* and *f_B2_* are BeiDou B_1_ and B_2_ signals frequencies.

(7) $$N_{G_{IF}}=\alpha_{G}\lambda_{1}N_{1}-\beta_{G}\lambda_{2}N_{2}$$

(8) $$N_{E_{IF}}=\alpha_{E}\lambda_{E1}N_{E1}-\beta_{E}\lambda_{E5a}N_{E5a}$$

(9) $$N_{B_{IF}}=\alpha_{B}\lambda_{B1}N_{B1}-\beta_{B}\lambda_{B2}N_{B2}$$

where *λ_1_* and *λ_2_* are the GPS L1 and L2 signals wavelengths in meters; *λ_E1_* and *λ_E5a_* are the Galileo E1 and E5a signals wavelengths in meters; *λ_B1_* and *λ_B2_* are the BeiDou B1 and B2 signals wavelengths in meters; *N_1_*, *N_2_* are the integer ambiguity parameters of GPS signals L1 and L2, respectively; *N_E1_*, *N_E5a_* are the integer ambiguity parameters of Galileo signals E1 and E5a, respectively; *N_B1_*, *N_B2_* are the integer ambiguity parameters of BeiDou signals B1 and B2, respectively.

Precise orbit and satellite clock corrections of IGS-MGEX networks are produced for GPS, Galileo and BeiDou observations and are referred to GPS time. IGS precise GPS satellite clock correction includes the effect of the ionosphere-free linear combination of the satellite hardware delays of L1/L2 P(Y) code, while the Galileo counterpart includes the effect of the ionosphere-free linear combination of the satellite hardware delays of the Galileo E1/E5a pilot code. In addition, BeiDou satellite clock correction includes the effect of the ionosphere-free linear combination of the satellite hardware delays of B1/B2 code [11]. By applying the precise clock products for GPS, Galileo, and BeiDou observations, Equations (1)–(6) will take the following form:

(10) $$P_{G_{IF}}=\rho_{G}+c[dt_{rG}-dt_{prec}^{s}]+c{\left[\alpha d_{P1}-\beta d_{P2}\right]}_{r}+T_{G}+\varepsilon_{PG_{IF}}$$

(11) $$P_{E_{IF}}=\rho_{E}+c[dt_{rG}-dt_{prec}^{s}]+c{\left[\alpha d_{E1}-\beta d_{E5a}\right]}_{r}+T_{E}+\varepsilon_{E_{IF}}$$

(12) $$P_{B_{IF}}=\rho_{B}+c[dt_{rG}-dt_{prec}^{s}]+c{\left[\alpha d_{B1}-\beta d_{B2}\right]}_{r}+T_{B}+\varepsilon_{B_{IF}}$$

(13) $$\Phi_{G_{IF}}=\rho_{G}+cdt_{rG}-c[dt_{prec}^{s}+{\left[\alpha d_{P1}-\beta d_{P2}\right]}^{s}]+c{\left[\alpha \delta_{L1}-\beta \delta_{L2}\right]}_{r}-c{\left[\alpha \delta_{L1}-\beta \delta_{L2}\right]}^{s}+T_{G}+N_{G_{IF}}+\phi_{r0_{G_{IF}}}+\phi_{0_{G_{IF}}}^{s}+\varepsilon_{\Phi G_{IF}}$$

(14) $$\Phi_{E_{IF}}=\rho_{E}+cdt_{rG}-c[dt_{prec}^{s}+{\left[\alpha d_{E1}-\beta d_{E5a}\right]}^{s}]+c{\left[\alpha \delta_{E1}-\beta \delta_{E5a}\right]}_{r}-c{\left[\alpha \delta_{E1}-\beta \delta_{E5a}\right]}^{s}+T_{E}+N_{E_{IF}}+\phi_{r0_{E_{IF}}}+\phi_{0_{E_{IF}}}^{s}+\varepsilon_{\Phi E_{IF}}$$

(15) $$\Phi_{B_{IF}}=\rho_{B}+cdt_{rG}-c[dt_{prec}^{s}+{\left[\alpha d_{B1}-\beta d_{B2}\right]}^{s}]+c{\left[\alpha \delta_{B1}-\beta \delta_{B2}\right]}_{r}-c{\left[\alpha \delta_{B1}-\beta \delta_{B2}\right]}^{s}+T_{B}+N_{B_{IF}}+\phi_{r0_{B_{IF}}}+\phi_{0_{B_{IF}}}^{s}+\varepsilon_{\Phi B_{IF}}$$

For simplicity, the receiver and satellite hardware delays are written as:
$b_{r_{P}}=c{\left[\alpha d_{P1}-\beta d_{P2}\right]}_{r}$$b_{P}^{s}=c{\left[\alpha d_{P1}-\beta d_{P2}\right]}^{s}$$b_{r_{E}}=c{\left[\alpha d_{E1}-\beta d_{E5a}\right]}_{r}$$b_{E}^{s}=c{\left[\alpha d_{E1}-\beta d_{E5a}\right]}^{s}$$b_{r_{B}}=c{\left[\alpha d_{B1}-\beta d_{B2}\right]}_{r}$$b_{B}^{s}=c{\left[\alpha d_{B1}-\beta d_{B2}\right]}^{s}$$b_{r_{\Phi }}=c{\left[\alpha \delta_{L1}-\beta \delta_{L2}\right]}_{r}+\phi_{r0_{G_{IF}}}$$b_{\Phi }^{s}=c{\left[\alpha \delta_{L1}-\beta \delta_{L2}\right]}^{s}+\phi_{{}_{0_{G_{IF}}}}^{s}$$b_{r_{E\Phi }}=c{\left[\alpha \delta_{E1}-\beta \delta_{E5a}\right]}_{r}+\phi_{r0_{E_{IF}}}$$b_{E\Phi }^{s}=c{\left[\alpha \delta_{E1}-\beta \delta_{E5a}\right]}^{s}+\phi_{{}_{0_{E_{IF}}}}^{s}$$b_{r_{B\Phi }}=c{\left[\alpha \delta_{B1}-\beta \delta_{B2}\right]}_{r}+\phi_{r0_{B_{IF}}}$$b_{B\Phi }^{s}=c{\left[\alpha \delta_{B1}-\beta \delta_{B2}\right]}^{s}+\phi_{{}_{0_{B_{IF}}}}^{s}$

In the combined GPS, Galileo and BeiDou un-differenced post-processed PPP solution, the GPS receiver clock error is lumped to the GPS receiver differential code biases. To maintain consistency in the estimation of a common receiver clock offset, this convention is used when combining the ionosphere-free linear combination of GPS L1/L2, Galileo E1/E5a, and BeiDou B1/B2 observations in the post-processed PPP solution. This, however, introduces an additional bias in the Galileo ionosphere-free PPP mathematical model, which represents the difference in the receiver differential code biases of both systems. Such an additional bias is commonly known as the inter-system bias, which is referred to as *ISB* in this paper. In our PPP model, the Hopfield tropospheric correction model along with the Vienna mapping function are used to account for the hydrostatic component of the tropospheric delay [27,31]. Other corrections are also applied, including the effect of ocean loading [32,33], Earth tide [28], carrier-phase windup [8,34], Sagnac [35], relativity [7], and satellite and receiver antenna phase-center variations [36]. The noise terms are modeled stochastically using an exponential model, as described in [37]. With the above consideration, the GPS/Galileo/BeiDou ionosphere-free linear combinations for the pseudorange and carrier-phase measurements can be written as:

(16) $$P_{G_{IF}}=\rho_{G}+\tilde{d}t_{rG}-dt_{prec}^{s}+T_{G}+\varepsilon_{PG_{IF}}$$

(17) $$P_{E_{IF}}=\rho_{E}+\tilde{d}t_{rG}-dt_{prec}^{s}+ISB_{GE}+T_{E}+\varepsilon_{E_{IF}}$$

(18) $$P_{B_{IF}}=\rho_{B}+\tilde{d}t_{rG}-dt_{prec}^{s}+ISB_{GB}+T_{B}+\varepsilon_{B_{IF}}$$

(19) $$\Phi_{G_{IF}}=\rho_{G}+\tilde{d}t_{rG}-dt_{prec}^{s}+T_{G}+{\tilde{N}}_{G_{IF}}+\varepsilon_{\Phi G_{IF}}$$

(20) $$\Phi_{E_{IF}}=\rho_{E}+\tilde{d}t_{rG}-dt_{prec}^{s}+T_{E}+{\tilde{N}}_{E_{IF}}+ISB_{GE}+\varepsilon_{\Phi E_{IF}}$$

(21) $$\Phi_{B_{IF}}=\rho_{B}+\tilde{d}t_{rG}-dt_{prec}^{s}+T_{B}+{\tilde{N}}_{B_{IF}}+ISB_{GB}+\varepsilon_{\Phi B_{IF}}$$

where $\tilde{d}t_{rG}$ represents the sum of the receiver clock error and receiver hardware delay $\tilde{d}t_{rG}=cdt_{rG}+b_{r_{P}}$; *ISB* is the inter system bias as follows $ISB_{GE}=b_{r_{E}}-b_{r_{P}}$; $ISB_{GB}=b_{r_{B}}-b_{r_{P}}$; ${\tilde{N}}_{G_{IF}}$, ${\tilde{N}}_{E_{IF}}$ and ${\tilde{N}}_{B_{IF}}$ are given by:

(22) $${\tilde{N}}_{G_{IF}}=N_{G_{IF}}+b_{r_{\Phi }}+b_{r_{P}}-b_{\Phi }^{s}-b_{P}^{s}$$

(23) $${\tilde{N}}_{E_{IF}}=N_{E_{IF}}+b_{r_{E\Phi }}+b_{r_{P}}-b_{E\Phi }^{s}-b_{E}^{s}$$

(24) $${\tilde{N}}_{B_{IF}}=N_{B_{IF}}+b_{r_{B\Phi }}+b_{r_{P}}-b_{B\Phi }^{s}-b_{B}^{s}$$

## 3. BSSD Post-Processing PPP Models

When combining the GPS, Galileo, and BeiDou observations in an un-differenced PPP model, the ambiguity parameters lose their integer nature as they are contaminated by the receiver and satellite hardware delays. It should be pointed out that the number of unknown parameters in the combined PPP solution equals the number of visible satellites from any system plus seven parameters, while the number of equations equals double the number of the visible satellites. This means that the redundancy equals *n_G_* + *n_E_* + *n_B_* − 7. In other words, at least seven mixed satellites are needed for the solution to exist. In comparison with the GPS-only un-differenced scenario, which requires a minimum of five satellites for the solution to exist, the addition of Galileo or BeiDou satellites increases the redundancy by *n_E_* + *n_B_* − 2. In other words, we need a minimum of three satellites from both Galileo and BeiDou systems in order to contribute to the solution.

As indicated earlier, the reference satellite can be selected from any of the three satellite constellations [37]. If a GPS satellite is selected as a reference for all GNSS observables, using Equations (16)–(21), BSSD mathematical models can be written as:

(25) $$\rho_{G,G}^{ij}+m_{f_{G,G}}^{ij}zpd_{w}^{}+{\tilde{\varepsilon }}_{PG_{IF}}^{ij}-{\tilde{P}}_{G_{IF}}^{ij}=0$$

(26) $$\rho_{E,G}^{ik}+m_{f_{E,G}}^{ik}zpd_{w}^{}+ISB_{GE}+{\tilde{\varepsilon }}_{PE_{IF}}^{ik}-{\tilde{P}}_{EG_{IF}}^{ik}=0$$

(27) $$\rho_{B,G}^{ih}+m_{f_{B,G}}^{ih}zpd_{w}^{}+ISB_{GB}+{\tilde{\varepsilon }}_{PB_{IF}}^{ih}-{\tilde{P}}_{BG_{IF}}^{iB}=0$$

(28) $$\rho_{G,G}^{ij}+m_{f_{G,G}}^{ij}zpd_{w}^{}+{\tilde{N}}_{G_{IF}}^{ij}+{\tilde{\varepsilon }}_{\Phi G_{IF}}^{ij}-{\tilde{\Phi }}_{G_{IF}}^{ij}=0$$

(29) $$\rho_{E,G}^{ik}+m_{f_{E,G}}^{ik}zpd_{w}+ISB_{GE}+{\tilde{N}}_{EG_{IF}}^{ik}+{\tilde{\varepsilon }}_{\Phi E_{IF}}^{ik}-{\tilde{\Phi }}_{EG_{IF}}^{ik}=0$$

(30) $$\rho_{B,G}^{ih}+m_{f_{B,G}}^{ih}zpd_{w}+ISB_{GB}+{\tilde{N}}_{BG_{IF}}^{ih}+{\tilde{\varepsilon }}_{\Phi B_{IF}}^{ih}-{\tilde{\Phi }}_{BG_{IF}}^{ih}=0$$

where ${\tilde{N}}_{{}_{G_{IF}}}^{ij}$, ${\tilde{N}}_{EG_{IF}}^{ik}$ and ${\tilde{N}}_{BG_{IF}}^{ih}$ are given by:

(31) $${\tilde{N}}_{{}_{G_{IF}}}^{ij}=N_{{}_{G_{IF}}}^{i}-N_{{}_{G_{IF}}}^{j}+b_{G\Phi }^{ij}-b_{P}^{ij}$$

(32) $${\tilde{N}}_{EG_{IF}}^{ik}=N_{{}_{G_{IF}}}^{i}-N_{{}_{E_{IF}}}^{k}+b_{r_{E\Phi }}-b_{r_{\Phi }}+b_{E\Phi }^{k}-b_{\Phi }^{i}+b_{P}^{i}-b_{E}^{k}$$

(33) $${\tilde{N}}_{BG_{IF}}^{ih}=N_{{}_{G_{IF}}}^{i}-N_{{}_{B_{IF}}}^{h}+b_{r_{B\Phi }}-b_{r_{\Phi }}+b_{B\Phi }^{h}-b_{\Phi }^{i}+b_{P}^{i}-b_{B}^{h}$$

Similarly, when a Galileo satellite is selected as a reference, using Equations (16)–(21), BSSD mathematical models can be written as:

(34) $$\rho_{G,E}^{lj}+m_{f_{G,E}}^{lj}zpd_{w}^{}-ISB_{GE}+{\tilde{\varepsilon }}_{PG_{IF}}^{lj}-{\tilde{P}}_{GE_{IF}}^{lj}=0$$

(35) $$\rho_{E,E}^{lk}+m_{f_{E,E}}^{lk}zpd_{w}^{}+{\tilde{\varepsilon }}_{PE_{IF}}^{lk}-{\tilde{P}}_{E_{IF}}^{lk}=0$$

(36) $$\rho_{B,E}^{lh}+m_{f_{B,E}}^{lh}zpd_{w}^{}+ISB_{GB}-ISB_{GE}+{\tilde{\varepsilon }}_{PB_{IF}}^{lh}-{\tilde{P}}_{BE_{IF}}^{lh}=0$$

(37) $$\rho_{G,E}^{lj}+m_{f_{G,E}}^{lj}zpd_{w}^{}-ISB_{GE}+{\tilde{N}}_{GE_{IF}}^{lj}+{\tilde{\varepsilon }}_{\Phi G_{IF}}^{lj}-{\tilde{\Phi }}_{GE_{IF}}^{lj}=0$$

(38) $$\rho_{E,E}^{lk}+m_{f_{E,E}}^{lk}zpd_{w}^{}+{\tilde{N}}_{E_{IF}}^{lk}+{\tilde{\varepsilon }}_{\Phi E_{IF}}^{lk}-{\tilde{\Phi }}_{E_{IF}}^{lk}=0$$

(39) $$\rho_{B,E}^{lh}+m_{f_{B,E}}^{lh}zpd_{w}^{}+ISB_{GB}-ISB_{GE}+{\tilde{N}}_{BE_{IF}}^{lh}+{\tilde{\varepsilon }}_{\Phi B_{IF}}^{lh}-{\tilde{\Phi }}_{BE_{IF}}^{lh}=0$$

where, ${\tilde{N}}_{GE_{IF}}^{lj}$, ${\tilde{N}}_{E_{IF}}^{lk}$, and ${\tilde{N}}_{BE_{IF}}^{lh}$ are the BSSD non-integer ambiguity parameters lumped to the receiver and satellite hardware delays, which are given by:

(40) $${\tilde{N}}_{GE_{IF}}^{lj}=N_{E_{IF}}^{l}-N_{G_{IF}}^{j}+b_{r_{G\Phi }}-b_{r_{E\Phi }}+b_{G\Phi }^{j}-b_{E\Phi }^{l}+b_{E}^{l}-b_{P}^{j}$$

(41) $${\tilde{N}}_{E_{IF}}^{lk}=N_{E_{IF}}^{l}-N_{E_{IF}}^{k}+b_{E\Phi }^{lk}-b_{E}^{lk}$$

(42) $${\tilde{N}}_{BE_{IF}}^{lh}=N_{E_{IF}}^{l}-N_{B_{IF}}^{h}+b_{r_{B\Phi }}-b_{r_{E\Phi }}+b_{B\Phi }^{h}-b_{E\Phi }^{l}+b_{E}^{l}-b_{B}^{h}$$

When selecting a BeiDou satellite as a reference, using Equations (16)–(21), BSSD mathematical models can be written as:

(43) $$\rho_{G,B}^{hj}+m_{f_{G,B}}^{hj}zpd_{w}^{}-ISB_{GB}+{\tilde{\varepsilon }}_{PG_{IF}}^{hj}-{\tilde{P}}_{GB_{IF}}^{hj}=0$$

(44) $$\rho_{E,B}^{hk}+m_{f_{E,B}}^{hk}zpd_{w}^{}+ISB_{GE}-ISB_{GB}+{\tilde{\varepsilon }}_{PE_{IF}}^{hk}-{\tilde{P}}_{EB_{IF}}^{hk}=0$$

(45) $$\rho_{B,B}^{hu}+m_{f_{B,B}}^{hu}zpd_{w}^{}+{\tilde{\varepsilon }}_{PB_{IF}}^{hu}-{\tilde{P}}_{B_{IF}}^{hu}=0$$

(46) $$\rho_{G,B}^{hj}+m_{f_{G,B}}^{hj}zpd_{w}^{}-ISB_{GB}+{\tilde{N}}_{GB_{IF}}^{hj}+{\tilde{\varepsilon }}_{\Phi G_{IF}}^{hj}-{\tilde{\Phi }}_{GB_{IF}}^{hj}=0$$

(47) $$\rho_{E,B}^{hk}+m_{f_{E,B}}^{hk}zpd_{w}^{}+ISB_{GE}-ISB_{GB}+{\tilde{N}}_{BE_{IF}}^{hk}+{\tilde{\varepsilon }}_{\Phi E_{IF}}^{hk}-{\tilde{\Phi }}_{EB_{IF}}^{hk}=0$$

(48) $$\rho_{B,B}^{hu}+m_{f_{B,B}}^{hu}zpd_{w}^{}+{\tilde{N}}_{B_{IF}}^{hu}+{\tilde{\varepsilon }}_{\Phi B_{IF}}^{hu}-{\tilde{\Phi }}_{B_{IF}}^{hu}=0$$

where, ${\tilde{N}}_{GB_{IF}}^{hj}$, ${\tilde{N}}_{EB_{IF}}^{hk}$, and ${\tilde{N}}_{B_{IF}}^{hu}$ are the BSSD non-integer ambiguity parameters lumped to the receiver and satellite hardware delays, which are given by:

(49) $${\tilde{N}}_{GB_{IF}}^{hj}=N_{B_{IF}}^{h}-N_{G_{IF}}^{j}+b_{r_{G\Phi }}-b_{r_{B\Phi }}+b_{G\Phi }^{j}-b_{B\Phi }^{h}+b_{B}^{h}-b_{P}^{j}$$

(50) $${\tilde{N}}_{EB_{IF}}^{hk}=N_{B_{IF}}^{h}-N_{E_{IF}}^{k}+b_{r_{E\Phi }}-b_{r_{B\Phi }}+b_{E\Phi }^{k}-b_{B\Phi }^{h}+b_{B}^{h}-b_{E}^{k}$$

(51) $${\tilde{N}}_{B_{IF}}^{hu}=N_{B_{IF}}^{h}-N_{B_{IF}}^{u}+b_{B\Phi }^{hu}-b_{B}^{hu}$$

Under the assumption that the observations are uncorrelated and the errors are normally distributed with zero mean, the covariance matrix of the un-differenced observations takes the form of a diagonal matrix. The elements along the diagonal line represent the variances of the code and carrier phase measurements. In our solution, we consider the ratio between the standard deviation of the code and carrier-phase measurements to be 100. When forming BSSD, however, the differenced observations become mathematically correlated. This leads to a fully populated covariance matrix at any particular epoch.

## 4. Real-Time PPP Satellite Clock Corrections

IGS launched its real-time service (RTS) in April 2013, which provides the users with real-time satellite orbit and clock corrections. At present, the IGS RTS uses a network of 130 globally distributed real-time tracking stations (IGS, 2016). Generally, the IGS satellite orbit and clock corrections are available to users with a delay based on the stated accuracies of the corrections, which are intended to be used in the post-processed positioning mode, e.g., the final IGS orbit and clock corrections have a delay of about 14 days. The IGS produced “ultra rapid” precise satellite correction products, which can be used in near real-time and real-time positioning; however, the prediction part of these corrections are based on earlier observations and are significantly less accurate than the other IGS products [26,38].

The IGS RTS produces and publishes real-time GNSS orbit and clock corrections, which are streamed to users in the Radio Technical Commission for Maritime services (RTCM) format. The RTCM State Space Representation (SSR) format is capable of supporting sub-decimeter RT-PPP anywhere in the world. Currently, the RTS products are offered for the GPS, Galileo, and GLONASS constellations. Table 1 outlines the IGS RTS products, their formats and frequency [25,26].

In order to access the RTS-IGS data streams that contain the satellite orbit and clock corrections, an NTRIP client application must be used. The Bundesamt für Kartographie und Geodäsie (BKG) NTRIP Client (BNC) version 2.11.1 is used to access these data streams. BKG Ntrip Client (BNC) is an open source application that support a variety of GNSS positioning applications [38].

IGS01/IGC01 precise satellite orbit and clock corrections are computed using a single epoch GPS combination. The solution epochs in this product are completely independent of each other, which has the advantage that the full accuracy is available as soon as product generation starts. While the IGS02 precise satellite orbit and clock corrections is extracted from one of the incoming ultra-rapid solutions. Both of the IGS02 and IGS03 use Kalman filtering and require a few minutes to converge to their full accuracy. The major difference between IGS03 and IGS02 is that the former includes GNSS corrections in addition to GPS [39].

## 5. Results and Discussion

**Figure 1:**
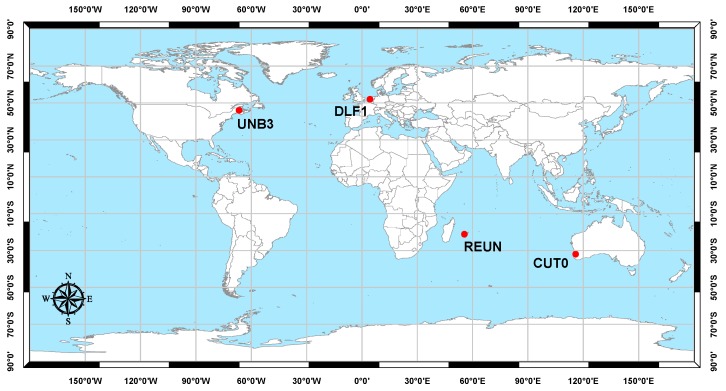
Analysis stations.

To verify the developed combined PPP model, three-constellation GNSS (GPS, Galileo, and BeiDou) observations at four globally distributed stations were selected from the IGS tracking network (Figure 1) [36]. Those stations are occupied by GNSS receivers, which are capable of simultaneously tracking the GNSS constellations. Only one hour of observations with maximum possible number of Galileo and BeiDou satellites at each station is considered in our analysis. All data sets have an interval of 30 s.

**Figure 2:**
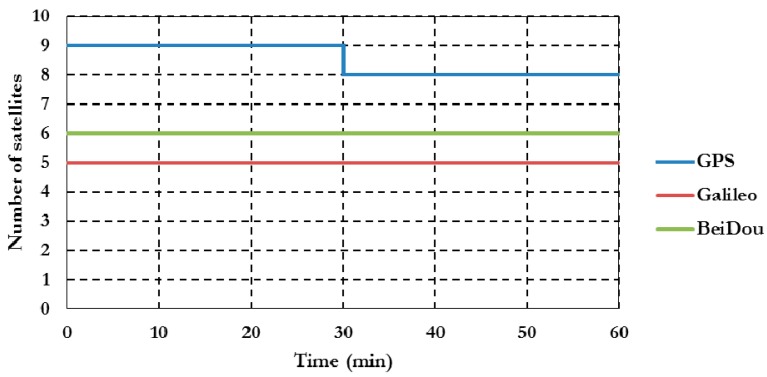
DLF1 station GNSS satellite availability.

The positioning results for station Delf1 located at Delft University of Technology, The Netherlands, are presented below. Similar results are obtained for the other stations. However, a summary of the convergence times and the three-dimensional PPP solution standard deviations are presented below for all stations. Natural Resources Canada’s GPSPace PPP software is modified to handle data from GPS, Galileo, and BeiDou systems, which enables a combined PPP solution as detailed above. BNC version 2.11.1 software is used to combine GPS and Galileo observations to obtain a real-time PPP solution. In addition to the combined PPP solution, we also obtained the PPP solutions of the un-differenced ionosphere-free GPS-only, which is used to assess the performance of the newly developed PPP model. Figure 2 summarizes the satellite availability during the one-hour observation time for each constellation at DLF1 station.

As shown in Figure 2, eight to nine GPS satellites were visible during the one-hour observation time span. The addition of Galileo and BeiDou systems increase the number of visible satellites to 19–20.

**Figure 3:**
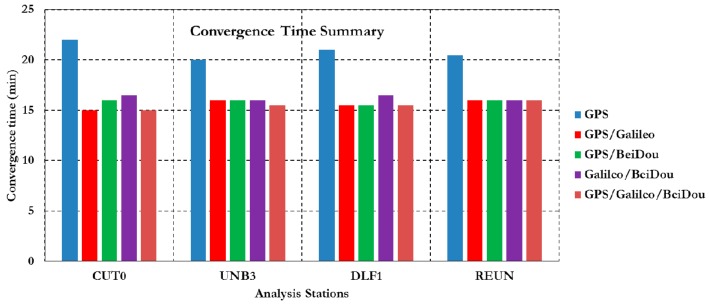
Summary of convergence times for post-processing PPP solutions at all stations.

**Figure 4:**
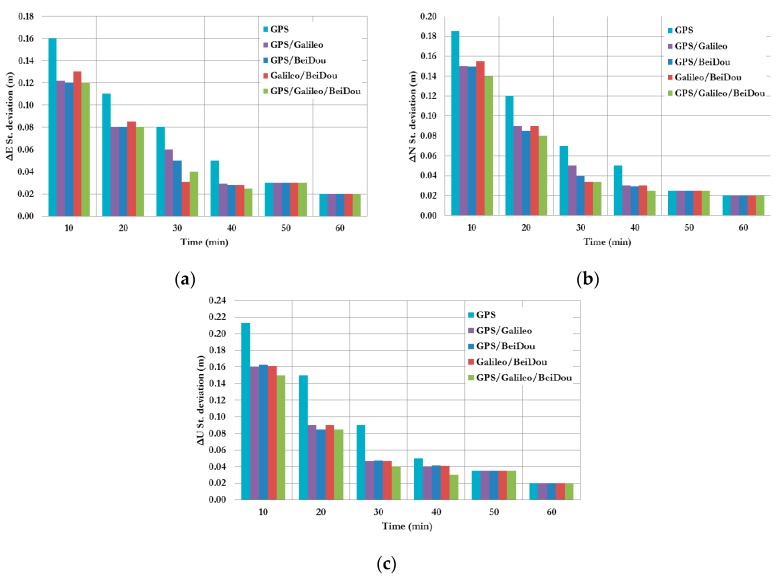
Summary of positioning standard deviations in East, North, and Up directions of all un-differenced post-processed PPP solutions. (**a**) East standard deviation; (**b**) North standard deviation; (**c**) Up standard deviation.

Figure 3 summarizes the convergence times for all un-differenced post-processing PPP models with different GNSS constellation combinations. As can be seen, the un-differenced GPS-only post-processed PPP solution indicates that the model is capable of obtaining a sub-decimetre level accuracy. However, the solution takes about 20 min to converge to decimetre level precision. As shown in Figure 3, the convergence time of the combined GNSS post-processed PPP solutions takes about 15 min to reach the decimeter level precision, which represent a 25% improvement in comparison with the GPS-only post-processed PPP solution. To further assess the performance of the various un-differenced post-processing PPP models, the solution output is sampled every 10 min and the standard deviation of the computed station coordinates is calculated for each sample. Figure 4 shows the position standard deviations in the East, North, and Up directions, respectively. As can be seen, the precision of the combined un-differenced post-processed PPP solutions is comparable to that of the GPS-only post-processed PPP solution.

**Figure 5:**
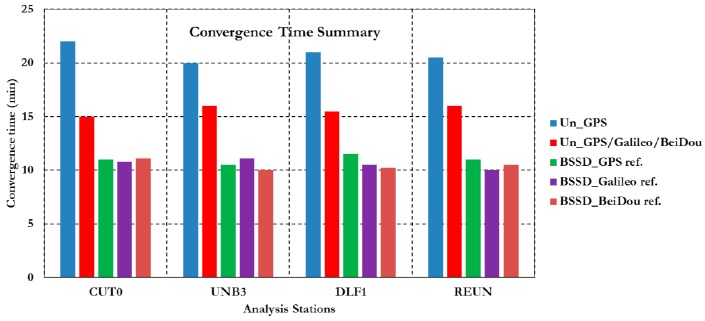
Summary of convergence times for BSSD post-processed PPP solutions.

Figure 5 summarizes the convergence times for the GNSS BSSD post-processed PPP solutions using different reference satellites. As shown in Figure 5, using BSSD post-processing PPP model reduces the convergence time to 10 min, which represents a 50% improvement compared to the GPS-only post-processed PPP solution. Similar to the un-differenced solution, the BSSD solution output is sampled every 10 min and the standard deviation of the estimated station coordinates is calculated for each sample.

**Figure 6:**
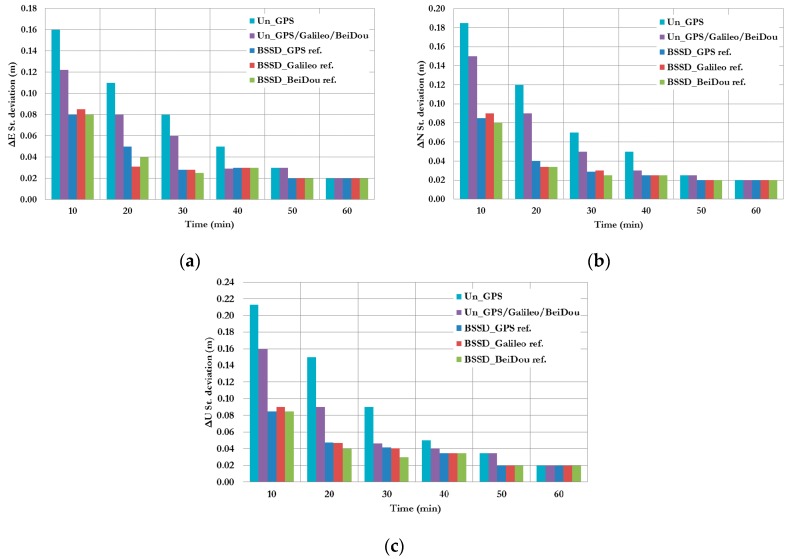
Summary of positioning standard deviations in East, North, and Up directions of all BSSD post-processed PPP solutions. (**a**) East standard deviation; (**b**) North standard deviation; (**c**) Up standard deviation.

Figure 6 shows a summary of the standard deviations of the station coordinates in the East, North, and Up directions, respectively. As shown in Figure 6, the standard deviations of the GNSS BSSD post-processed PPP solutions are improved compared to the un-differenced post-processed PPP solutions. In addition, as the number of epochs, and consequently the number of measurements, increases the performance of the various models tends to be comparable. In order to assess the RT-PPP solution, all of the IGS RTS products are used to produce various real-time PPP solutions.

**Figure 7:**
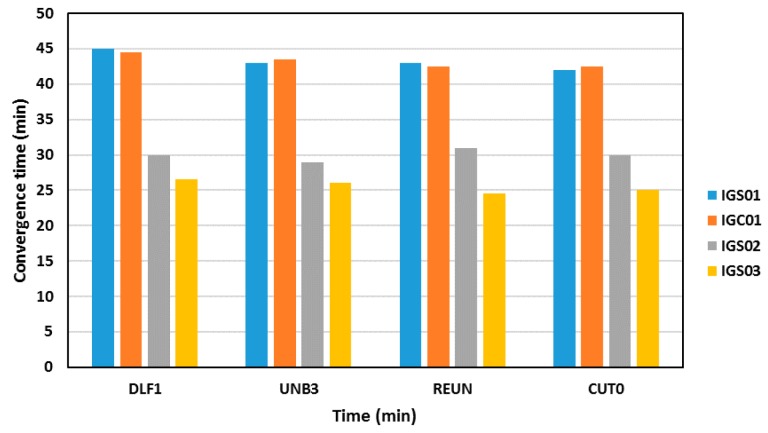
Summary of convergence times of RT-PPP solutions using different satellite clock products.

Figure 7 summarizes the convergence times of the various RT-PPP solutions. As shown in Figure 7, the RT-PPP solution convergence time depends on the IGS RTS satellite orbit and clock corrections used. The IGS03 satellite corrections provided the best PPP solution, with a convergence time around 25 min. This is expected because the IGS03 precise satellite products corrections are based on multi-constellation GNSS solution. The other RTS-IGS products provided longer PPP solution convergence times.

**Figure 8:**
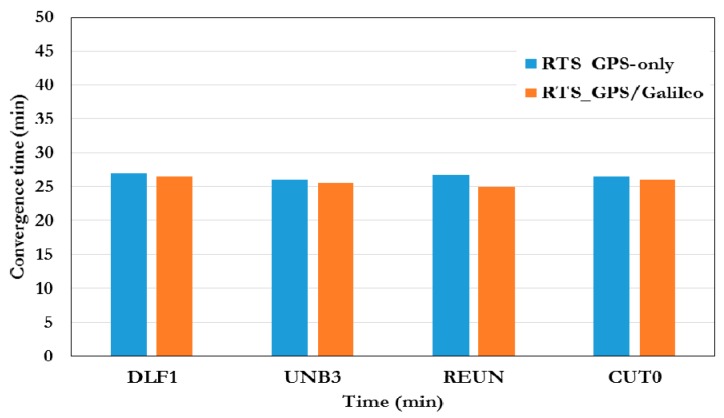
A summary of the convergence time of RT-PPP solutions using IGS03 satellite clock correction.

Figure 8 summarizes the convergence times of the RT-PPP solutions for the various test stations when the IGS03 satellite corrections are used. As can be seen, the convergence time for the RT-PPP solution is similar to the convergence time of the GPS-only RT-PPP solution. As mentioned earlier the RTS-IGS satellite clock corrections are not available for the BeiDou system. Similar to previous cases, the RT-PPP solution output is sampled every 10 min and the standard deviation of the estimated station coordinates is calculated for each sample.

**Figure 9:**
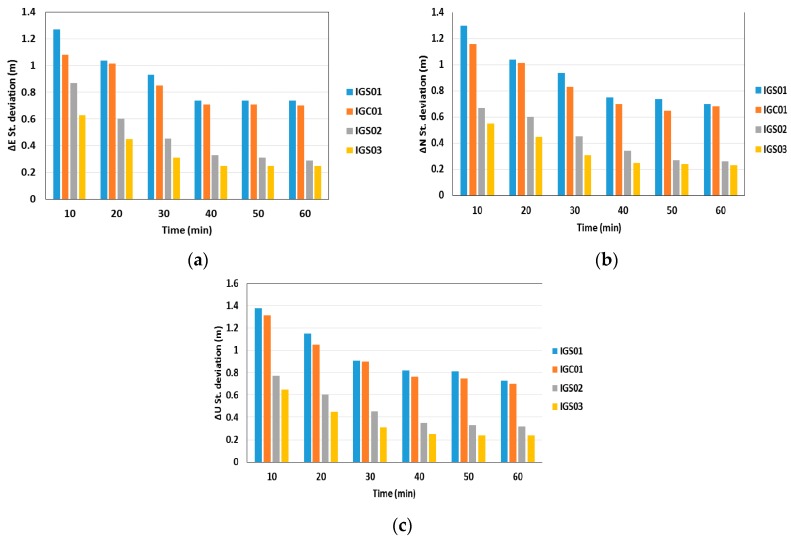
Summary of the RT-PPP positioning standard deviations in East, North, and Up directions. (**a**) East standard deviation; (**b**) North standard deviation; (**c**) Up standard deviation.

Figure 9 shows the standard deviations of the station coordinates in the East, North, and Up directions, respectively. As can be seen, the use of IGS03 satellite corrections improves the RT-PPP solution precision, in comparison with other real-time satellite correction products.

## 6. Conclusions

This paper developed a triple-constellation GNSS PPP mode in various positioning modes. Post-processed PPP solutions are obtained in both of the un-differenced and BSSD modes, while a RT-PPP solution is obtained the un-differenced mode only. All post-processed PPP and RT-PPP solutions are compared with the traditional un-differenced GPS-only counterparts. Three scenarios are considered when forming BSSD, each uses a reference satellite from a different GNSS constellation. All of the RTS-IGS satellite corrections currently produced by the IGS are used to produce the RT-PPP solutions.

It has been shown that combining the GPS, Galileo, and BeiDou observations in an un-differenced post-processing PPP model improves the convergence time by about 25% in comparison with the GPS-only counterpart. However, no noticeable improvement is obtained in the PPP solution precision. The use of BSSD linear combination improves the PPP solution convergence time by about 50% and the precision of the estimated PPP parameters by about 25%, in comparison with the GPS-only post-processed PPP solution. RTS IGS03 satellite corrections provided the shortest RT-PPP convergence time, which was about 25 min. In addition, the precision of the RT-PPP solution was better when IGS03 was used, in comparison with the cases when IGS01, IGC01, and IGS02 satellite corrections were used.

## Figures and Tables

**Table 1:** Outlines the IGS RTS products, their formats and frequency.

Product	Format	Frequency
GNSS Data	RTCM 3	1 s
GPS orbit corrections	RTCM-SSR	5 or 60 s
GPS clock Corrections	RTCM-SSR	5 s
